# Development of Intelligent Indicators Based on Cellulose and *Prunus domestica* Extracted Anthocyanins for Monitoring the Freshness of Packaged Chicken

**DOI:** 10.1155/2024/7949258

**Published:** 2024-03-28

**Authors:** Mustafa Ahmed, Ipsheta Bose, Nousheen, Swarup Roy

**Affiliations:** ^1^School of Bioengineering and Food Sciences, Shoolini University, Solan 173229, India; ^2^Department of Food Technology and Nutrition, School of Agriculture, Lovely Professional University, Phagwara 144411, India

## Abstract

Meat is a widely consumed food globally; however, variations in storage conditions along its supply chain can pose a potential food safety risk for consumers. Addressing this concern, we have developed freshness indicators designed to monitor the condition of packaged chicken. In this study, anthocyanins were infused with cellulose paper measuring 2 × 2 cm, and subsequent analysis focused on examining color changes concerning deteriorating chicken stored at 30°C for 48 h, with varying sample sizes being considered. The rise in total volatile nitrogen (TVB-N) compounds from an initial value of 3.64 ± 0.39 mg/100 g to 28.17 ± 1.46 mg/100 g acted as the stimulus for the color change in the indicator, simultaneously influencing the pH from the initial 7.03 ± 0.16 to 8.12 ± 0.39. The microbial load (aerobic plate count) of the chicken samples was also significantly increased. This collective shift in various parameters strongly suggests the occurrence of spoilage in chicken meat. The pH indicators exhibited a dark pink to red color for fresh chicken. As the chicken meat turned towards spoilage, the indicators changed to a dark blue and then a pale green color. FTIR spectroscopy results confirmed the presence of cellulose and anthocyanins. The FTIR analysis also validated the immobilization of plum anthocyanins within the cellulose paper and assessed their stability after 8 months of storage. Notably, the indicators demonstrated rapid sensitivity, showing a 20.5% response within one minute of ammonia exposure, which further increased to 29.5% after 3 min of exposure. The total color difference (ΔE) steadily rose in all the examined samples and also under various storage conditions. Overall, the indicators developed in this study exhibited a highly pronounced color transition, capable of distinguishing between fresh and spoiled chicken samples depending on the extent of spoilage and the specific day of observation.

## 1. Introduction

Poultry meat is the most widely consumed meat globally, but it undergoes spoilage within one week after being slaughtered, regardless of the refrigeration method used. This spoilage is caused by a variety of microorganisms, including bacteria such as *Shewanella putrefaciens* and *Pseudomonas* spp., along with yeast [1]. However, the specific types of microorganisms responsible can vary based on the initial microbiological condition of the carcass [2]. This meat spoilage poses a food safety risk for consumers within the poultry meat supply chain. Therefore, a strong demand exists among various stakeholders in the food industry, to create precise, cost-effective, fast, dependable, and nonintrusive methods or devices for monitoring the current freshness status of meat products in real time [3]. The emergence of intelligent indicators for food spoilage is being considered as an alternative to meet this consumer demand. These indicators are designed to continuously evaluate and monitor the freshness of meat and other food products [4]. Among these, there are color-changing indicators that react to external factors, such as moisture, gases, electromagnetic radiation, or temperature, and display visible color changes [5]. These color shifts can be associated with ongoing changes in the quality of the food product. Such interactive packaging systems enable the constant monitoring of product freshness throughout transportation, distribution, storage, and marketing [6]. These color indicators work on the concept that when microbes grow on packaged food items, their metabolites interact with the indicators, leading to a visible color change due to shifts in pH levels [7]. Essentially, the freshness indicator responds to the metabolites generated by microbial growth in packaged foods and undergoes color changes due to pH fluctuations [8]. When protein in meat is decomposed or denatured, it produces large amounts of volatile organic amines, which results in an increase of pH in the headspace of the meat packaging. By using this design concept, the pH-responsive color-changing indicators could be applied in the development of an intelligent food packaging system to visually identify spoilage or indicate the loss of freshness of meat and other food items. These indicators allow consumers to assess the freshness or quality of the food based on chemical alterations or microbial growth that occurs during the distribution process [9].

The pH-based smart indicator includes two components: a base such as a polymer matrix and a dye that responds to pH levels. Many of the dyes currently employed in intelligent food packaging are synthetic and as a result carry toxic properties, with some of them even being considered carcinogenic, posing significant risks to both human health and the environment [10]. Consequently, there is a pressing need to substitute these hazardous dyes with renewable, nontoxic natural pigments. Therefore, the adoption of natural colorants is eco-friendly and nontoxic and can enhance food safety and align with consumer expectations for safer food products [11]. Among these alternatives, anthocyanin stands out as the most widely utilized pigment due to its ability to change color over a wide pH range, safety profile, and abundant availability, with additional benefits such as antimicrobial and antioxidant properties [12]. It can be utilized to monitor food quality, signal the shelf life of products, pique consumer interest in food items, and ultimately serve as a smart color indicator for food packaging applications [13, 14].

In this study, anthocyanins from common plum (*Prunus domestica*) were used to create a cellulose-based freshness indicator for monitoring raw chicken spoilage. This innovative approach offers a promising solution to ensure the safety and quality of the meat. The intelligent indicators effectively differentiate between fresh and spoiled chicken at various stages, enhancing quality control in the food processing industry. The acylated anthocyanins from *Prunus domestica* contribute to the storage efficiency and shelf life, making them cost-effective and practical for continuous monitoring. This research advances the field of intelligent packaging, providing a reliable method to enhance food safety and quality.

## 2. Materials and Methods

### 2.1. Materials

All chemicals used in this experiment were sourced from Loba Chemie Pvt. Ltd., Mumbai, India.

These include 99.8% methanol (AR grade), 1N hydrochloric acid (HCl) solution, 98% sodium hydroxide (NaOH) pellets (AR grade), 25% extra-pure ammonia solution, and 90% extra-pure acetic acid.

### 2.2. Extraction of Anthocyanins

Ripe fruits of plum (*Prunus domestica*) were harvested from the local farm of Shili [ 30.9090°N, 77.1512°E], rural Shimla, Himachal Pradesh, India, in the month of May. The fruits were promptly conveyed to the lab, placed in plastic bags, and stored in a refrigerator at temperatures below 4°C. The fresh fruits were then rinsed under running tap water and dried, and thin peels were obtained. 20 g peels were directly ground in the blender with 40 mL acidified methanol (0.01% (v/v) HCl in methanol. The obtained slurry was allowed to be macerated overnight under refrigerated conditions. The mixture was passed through a Whatman no. 1 filter paper. The plant material was subjected to additional acidified methanol extraction until a lightly colored extract was obtained. The pool was filtered, and plant material was discarded. The filtered liquid was then transferred into a boiling flask and subjected to evaporation of the solvent in a rotary evaporator (Hei-VAP Core-G3 vertical glassware rotary evaporator, Heidolph Instruments, Germany) at 40°C under vacuum. The total monomeric anthocyanin content as determined by the pH differential method was 102 mg/L of the extract [15]. The remaining concentrated aqueous extract was kept at 4°C in the refrigerator.

### 2.3. pH-Dependent Color Change and UV-Vis Spectral Analysis

The alteration in color of the methanolic plum anthocyanin extract (PA) was observed across a range of pH values, spanning from 2 to 12. To conduct this experiment, six distinct pH buffer solutions (2, 4, 6, 7, 9, and 12) were prepared by adjusting the pH using NaOH and HCl in distilled water. Next, 10 mL of each pH solution was distributed into six separate test tubes, and labeling them accordingly. Subsequently, 1 mL of the PA was added to each test tube, and the resulting color changes were observed. To analyze these differently colored solutions, their light absorption spectra were measured using Evolution 201 UV-Visible spectrophotometer (Thermo Fisher Scientific, Waltham, Massachusetts, US) within the wavelength range of 300–700 nm.

### 2.4. Preparation of Intelligent Indicators

In this research, standard qualitative filter paper (used for conventional filtration) with a pore size of 12–15 *μ*M and thickness of 180 *μ*m composed of cellulose, served as the base for the indicator. The paper was cut into 2 × 2 cm pieces. On each of these paper strips, a 2 mL concentrated aqueous solution of plum anthocyanin, equivalent to 0.2 mg of anthocyanin per indicator strip, was applied. Subsequently, these indicators were stored in both dry and refrigerated conditions.

### 2.5. FTIR Analysis

To evaluate the chemical composition and confirm the existence of anthocyanins within the cellulose filter paper, Fourier transform infrared spectroscopy (FTIR) analysis was conducted on cellulose paper, PA, and the prepared intelligent indicators. To evaluate the shelf life of these indicators, FTIR was performed on each indicator at various stages: when fresh, at 2 months old, and at 8 months old. Additionally, the analysis was carried out for each indicator at both the 24-hour and 48-hour marks, using different meat sample sizes (50 g, 250 g, and 500 g) in the packaging test to assess alterations in the chemical composition of the indicators and their efficiency with samples of different sizes. This analysis was carried out using the Cary 360 FTIR spectrometer (Agilent Technologies, USA) within the spectral range of 4000–650 cm^−1^. The film samples were examined using attenuated total reflectance (ATR) mode.

### 2.6. Sensitivity to Ammonia

The sensitivity of the intelligent indicators to ammonia was assessed following the earlier published method [16]. To conduct this assessment, 80 mL of a 25% extra-pure ammonia solution (Loba Chemie Pvt. Ltd., Mumbai, India) was placed in a beaker, leaving approximately 1 cm of headspace between the solution's surface and the top of the beaker. A piece of the indicator was positioned at a distance of 1 cm from the solution's surface. Color changes of the indicator were captured through photographs taken at 3-minute intervals. Following that, the Pixie program was used to capture the RGB (Red, Green, and Blue) values of the indicator. The indicator's responsiveness to ammonia was calculated using the subsequent equation:

(1) $$\begin{array}{c} ∆R=\left|R_{a}\;–\;R_{b}\right|\\ ∆G=\left|G_{a}\;–\;G_{b}\right|\\ ∆B=\left|B_{a}\;–\;B_{b}\right|\\ s_{\text{RGB}}=\frac{∆R+∆G+∆B}{R_{a}+G_{a}+B_{a}}\times 100\;\%, \end{array}$$

*R*_*a*_, *G*_*a*_, and *B*_*a*_ values represent the RGB values of the indicator before exposure, while *R*_*b*_, *G*_*b*_, and *B*_*b*_ values represent the RGB values of the indicator after being exposed to an ammonia solution.

### 2.7. Food Sensing Application of the Intelligent Indicators

The evaluation of pH-sensitive freshness indicator activity was carried out using chicken meat. To do this, fresh chicken was procured from the market and transported to the laboratory under refrigerated conditions. Chicken samples of different sizes 50 g, 250 g, and 500 g were kept in polyethylene terephthalate (PET) containers, and the prepared indicator was attached to the interior of the PET lid. After sealing the packaging, the chicken was spoiled at 30 °C for 48 hours until visible signs of spoilage became apparent. To assess the progression of spoilage, samples were inspected at various time intervals during storage, such as day 0 (fresh), day 1 (spoiled for 24 hours), and day 3 (spoiled for 48 hours). For this purpose, 10 grams of chicken sample was completely blended in 90 mL of distilled water, filtered, and the pH was measured with a digital pH meter (Microprocessor pH mV Temperature Meter, Systonic Systems, India). The total volatile basic nitrogen (TVB-N) of the chicken samples was determined at day 0, day 1, and day 2 by the Conway microdiffusion method [17]. Consequently, the microbial load of the samples at all three stages was determined as APC (aerobic plate count) with plate count agar (PCA) as the media. The incubation time and temperature were 35°C and 48 h, respectively [18].

### 2.8. Optical Properties

The color attributes of the indicators were assessed at two distinct stages: initially when the indicators were in a fresh state, and subsequently, after they had been exposed to spoiling chicken for 48 hours during the packaging test. During this examination, colorimetric parameters, such as redness, blueness, yellowness, and whiteness, were determined using a Lovibond® Model F Colorimeter (Tintometer® Group, Lovibond House, UK). The visual colorimeter was set up with two neighboring fields of view visible through the viewing tube. This arrangement allowed for the simultaneous observation of an indicator in the sample field and a white reflective surface in the comparison field, both appropriately illuminated. Lovibond® glasses were incorporated into the comparison field using a system of sliding racks. This system permitted the comparison of the color of light transmitted through or reflected from the sample. The racks were adjusted until a visual color match was achieved between the light from the sample, and the corresponding Lovibond® units were used to express its color. Additionally, the indicator's color alterations were documented by capturing photographs of each indicator at both stages. The total color difference (ΔE) of the indicators was calculated using the following equation:

(2) $$\begin{array}{c} ∆E=\sqrt{\left({\left(L0*-{\;L}^{*}\right)}^{2\;}+{\left(a0*-a*\right)}^{2\;}+\left(b0*-b*\right)2\right)}, \end{array}$$

*L*^*∗*^, *a*^*∗*^, and *b*^*∗*^ represent the color characteristics of the indicators at different pH levels, whereas *L*_0_*∗*, *a*_0_*∗*, and *b*_0_*∗* denote the color parameters of the indicators in their initial condition.

### 2.9. Statistical Analysis

Repeated experiments were conducted, and the data represented as the mean and standard deviation of at least three observations. The experiment results were analyzed by one-way analysis of variance (ANOVA) by Microsoft Excel 2016.

## 3. Results and Discussions

### 3.1. pH-Dependent Color Change and UV-VIS Spectroscopic Analysis

**Figure 1:**
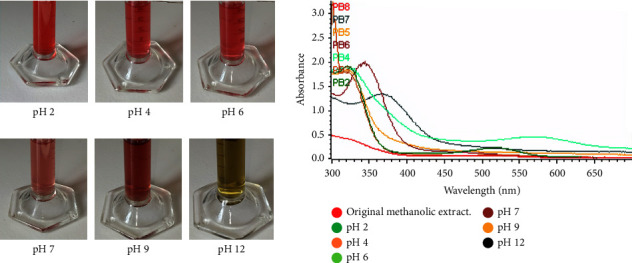
Color change of methanolic plum (*Prunus domestica*) anthocyanin extract at different pHs (2, 4, 6, 7, 9, and 12) and their UV-vis spectra.

**Figure 2:**
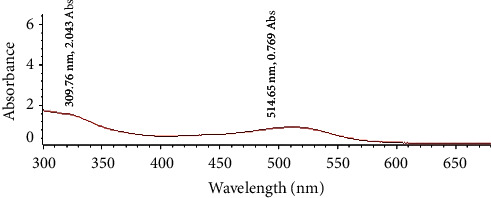
UV-vis spectrum of methanolic plum (*Prunus domestica*) anthocyanin extract.

The pH-dependent color change of PA is shown in Figure 1. Anthocyanins exhibit structural alterations based on pH levels.  Figure 2 displays the recorded color shift of PA within the pH range of 2 to 12. In the presence of various pH solutions, the PA solution displayed distinctive colors: a vivid red at pH 2, a pinkish red between pH 4 and 6, a purplish-brown within the pH range of 7 to 9, and a bright green at pH 12. The variation in PA's color in diverse pH solutions can be attributed to the transformation of their structure, transitioning from a red flavylium cation (pH < 5) to a blue/purple quinoidal anhydrobase (pH 6–9) and eventually to a green chalcone (pH > 10) [19, 20]. The UV-Vis spectroscopy results of the various anthocyanins containing pH solution are shown in Figure 2, and it displayed two primary absorbance peaks. The first peak, occurring at a wavelength of 515 nm, is connected with the existence of the flavylium cation. This pattern of absorption is a common characteristic, with maximum absorption (*λ*_max_) typically falling within the range of 510–520 nm in the visible spectrum. Additionally, there is another peak close to 310 nm, which is only present when the sugar component is acylated. This peak is associated with the carbinol pseudobase and chalcone. Notably, in nonacylated anthocyanins, this peak is either significantly reduced or appears as a minor elevation [21]. The absence of peaks within the 300–330 nm range in Chinese plum (*Prunus salicina*) anthocyanin indicates that the common plum (*Prunus domestica*) used in this study could be a more suitable candidate for creating color-based indicators. Moreover, the color of the extracted anthocyanin was stable for several months under dark and sealed conditions, with only slight fading in color perceived with more storage time. This is because the acyl groups in common plum's anthocyanin contribute to enhanced stability, particularly in terms of resistance to light and temperature variations [22].

### 3.2. FTIR Analysis

**Figure 3:**
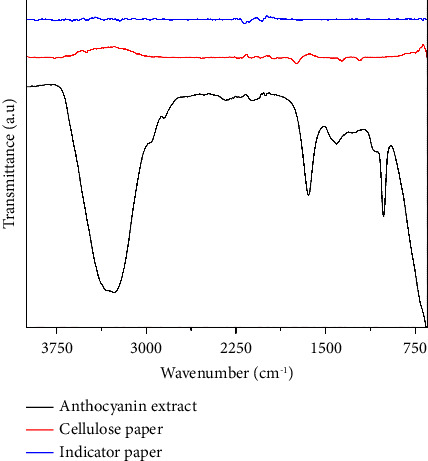
FTIR spectra of plum anthocyanin extract, cellulose paper, and the fabricated indicator.

The cellulose-based filter paper spectrum is presented in Figure 3. The FTIR peaks were identified within the wavenumber range of 3600−2900 cm⁻^1^, representing the stretching vibrations of O-H and C-H bonds found in polysaccharides. Notably, the broad peak at 3508 cm⁻^1^ was a distinctive feature associated with the stretching vibration of hydroxyl groups in polysaccharides, as reported earlier [23]. The band at 1752 cm⁻^1^ corresponds to the presence of C=O. Additionally, the band observed at 1380 cm⁻^1^ could be attributed to the CH_2_ bending vibration associated with the syringyl and guaiacyl rings in the lignin component of the filter paper. Meanwhile, the presence of pectin content is characterized by an absorption peak of carbonyl groups at 1752 cm⁻^1^ [24]. The spectrum of plum anthocyanins is shown in Figure 3. The peaks at 3270 cm⁻^1^, 2853 cm⁻^1^, and 1490 cm⁻^1^ were indicative of -OH stretching, C-H stretching, and -OH bending, respectively [25]. The bands observed at 1648 cm⁻^1^ were associated with the presence of C=C groups within aromatic rings. The absorbance recorded at 1022 cm⁻^1^ was a result of the stretching vibration of C-O-C esters [26, 27]. Consequently, the spectral examination of the concentrated extracts indicates the existence of O-H, C=C, and C-O-C functional groups, which are typical features of anthocyanins. In the FTIR analysis of the indicator (Figure 3), the O-H stretching band exhibited a downward shift to lower wavenumbers (3233 cm⁻^1^, 3263 cm⁻^1^, 3360 cm⁻^1^, and 3493 cm⁻^1^) when compared to the corresponding peaks in the cellulose-based filter paper (3650 cm⁻^1^ and 3508 cm⁻^1^) and plum anthocyanin (3270 cm⁻^1^). The similar shift observed in the characteristic band, previously at 1752 cm⁻^1^ (in cellulose paper) and 2853 cm⁻^1^ (in plum anthocyanins), to 1722 cm⁻^1^ and 2816 cm⁻^1^ in the indicator, implies that the incorporation of plum anthocyanins within the cellulose-based filter paper was accomplished through the formation of hydrogen bonds [25].

**Figure 4:**
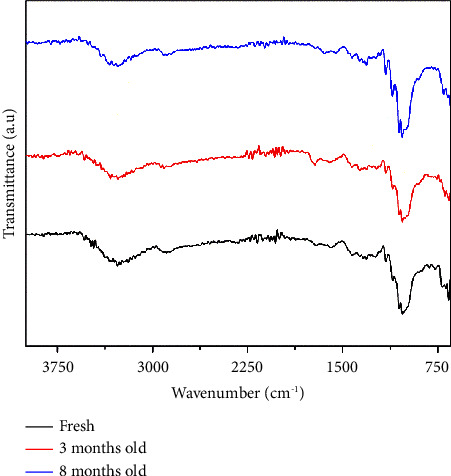
FTIR spectra of the fresh, 3-month-old, and 8-month-old pH-responsive color indicator.

**Figure 5:**
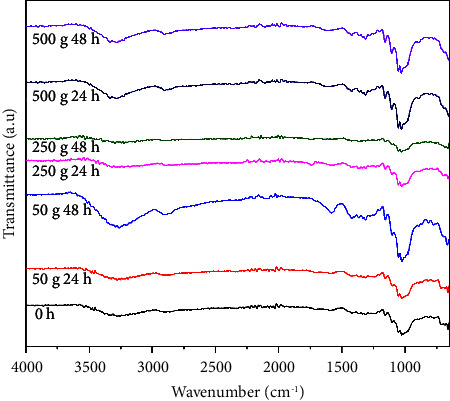
FTIR spectra of color indicator performance with different stages of chicken spoilage (0 h, 24 h, and 48 h) and sample sizes (50 g, 250 g, and 500 g).

The two-month old and eight-month old showed consistent absorption bands at 1428 cm⁻^1^, 1372 cm⁻^1^, 1339 cm⁻^1^, and 1033 cm⁻^1^ (Figure 4), aligning with the stretching and bending vibrations of -CH_2_ and -CH, -OH, and C-O bonds in cellulose [28, 29]. The broad peak at 1428 cm⁻^1^ encompasses vibrations of -CH_2_ and CH bonds from cellulose, along with the vibration of C = O bonds in the carbonate ion (CO_3_^2−^) [30]. The bands detected in the two-month-old indicator, ranging from 1428 cm⁻^1^ to 1722 cm⁻^1^ in the FTIR spectrum, confirmed the presence of a benzene ring (C = C/C = N/C = O) [31]. In the eight-month-old indicator, sharp bands at 1448 cm⁻^1^ indicate C=C, while 1033 cm⁻^1^ corresponds to C-O-C esters [26, 27]. In both indicators' spectra, bands at 3275 cm⁻^1^, 3335 cm⁻^1^, 3540 cm⁻^1^, 3309 cm⁻^1^, and 3279 cm⁻^1^ were associated with H-bonds, collectively suggesting the existence of –OH, C=C, and C-O-C groups indicative of anthocyanins [32]. This analysis underscores the stable nature of acylated plum anthocyanins in the cellulose indicator, especially when stored in dark conditions. Furthermore, similar patterns of cellulose and anthocyanin integration were noted in the spectra of indicators across various sample sizes and deterioration stages as shown in Figure 5.

### 3.3. Sensitivity to Ammonia

**Figure 6:**
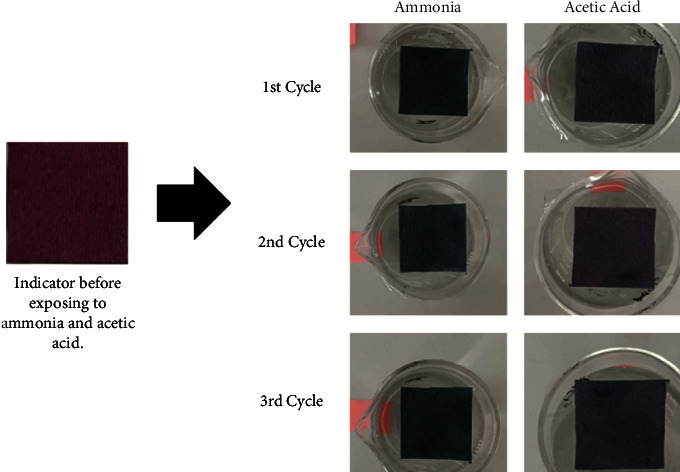
Cellulose-based indicator's color change in the presence of ammonia vapor and reversibility on exposure to acetic acid.

**Figure 7:**
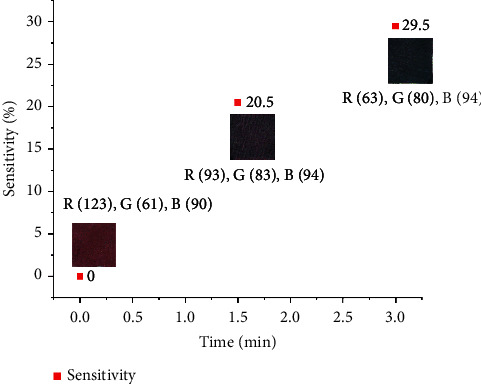
RGB value data of indicators as per the pixie program and their time-sensitivity graph with respect to ammonia.

The color change of intelligent indicators depends on their sensitivity to certain acidic/basic compounds such as ammonia and acetic acid. This test was performed as a preliminary examination of the indicator's color change in the presence of volatile gaseous substances, similar to those released when protein-rich foods spoil. Upon subjecting the indicators to ammonia vapor, a significant alteration in color was evident. On 1.5 min exposure, the color changed from pinkish red (original) to light blue. On further exposure of 3 min, a dark blue color was developed (Figure 6). This experiment demonstrated a prompt response of the prepared indicators to ammonia. The ammonia sensitivity was determined using the procedure outlined in the previously published report [16]. On 1.5 min exposure, the sensitivity was 20.5% whereas, on 3 min exposure, it was 29.5%.  Figure 7 shows the RGB (Red, Green, and Blue) values data of the indicators in the Pixie program with a graph depicting their sensitivity to ammonia. The swift alteration in the color of these indicators when exposed to ammonia could be attributed to the existence of polyphenolic compounds within anthocyanins and their structural alteration because of hydroxyl ions' interaction and the generation of oxygen anions. The presence of ammonium hydroxide ions resulting from the hydrolysis of ammonia creates an alkaline environment within the indicators [33]. Consecutively, when the indicators were placed in the presence of acetic acid, the color transformation was reverted which shows the reversibility of these indicators to color change. Previous research has already documented that a freshness indicator comprising bacterial nanocellulose anthocyanins exhibited a substantial sensitivity to ammonia, reaching 67% sensitivity after a 4-minute exposure [25]. However, in a similar investigation, a freshness indicator utilizing grape anthocyanin combined with cellulose demonstrated a sensitivity of 29.9% after 3 minutes of contact [34].

### 3.4. Food Application of Smart Indicators

**Figure 8:**
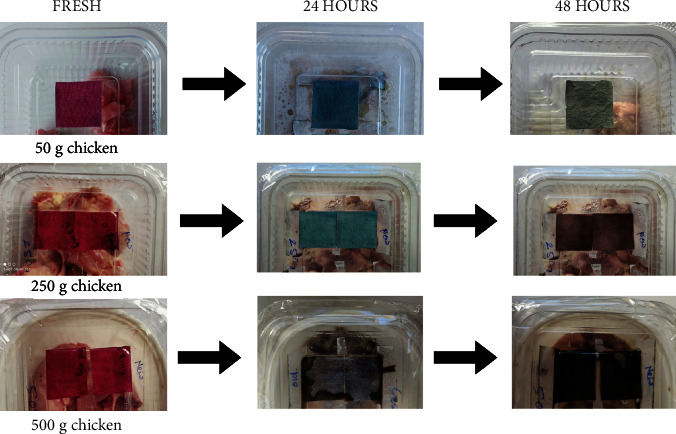
Color change of intelligent indicators with spoilage of packaged chicken of different sample sizes (50 g, 250 g, and 500 g) and days of storage (day 0, day 1, and day 2).

Meat products are prone to deterioration because of microbial processes that generate carbon dioxide and nitrogen-containing volatile compounds such as dimethylamine, trimethylamine, and ammonia [35]. These volatile compounds get collected in the container headspace which allows the freshness indicators to change their color and monitor the spoilage of packaged meat. To determine the extent of spoilage, meat samples were collected at different stages, namely, day 0 (fresh), day 1 (24 h), and day 2 (48 h), and their TVB-N values, pH, and microbial load (APC) were determined, as outlined in Table 1. The TVB-N value for fresh chicken meat was 3.64 ± 0.39 mg/100, indicating freshness. However, by day 1, it escalated to 13.57 ± 0.56 mg/100 g, approaching spoilage, given that the critical TVB-N range is set at 15–25 mg/100 g [36]. On day 2, the value exceeded the critical limit, with a TVB-N of 28.17 ± 1.46 mg/100 g, indicating the meat is unfit for consumption. The pH of the samples exhibited an upward trend, starting from 7.03 ± 0.16 when fresh, reaching 7.72 ± 0.08 on day 1, and further increasing to 8.12 ± 0.39 on day 2. Concurrently, the microbial load, measured as APC, was substantially increased to unacceptable levels from day 1 onwards (Table 1). Corresponding to these changes, with sample sizes of 50 g and 250 g, the indicators retained their original pinkish red color on day 0 of spoilage, transforming to dark blue on day 1. Upon further spoilage (day 3), the indicator with a 50 g chicken sample changed from blue to pale green, while the one with a 250 g chicken sample appeared brownish, most likely due to higher TVB-N amounts in the container headspace (Figure 8). Furthermore, the indicator with a sample size of 500 g showed varied results—light brown on day 1 and a dark blackish-brown color on day 2. This variation in the color of the indicator could be attributed to both high TVB-N content and moisture accumulation in the container headspace.

In a similar study, a film developed using plum peel anthocyanins showed a noticeable alteration in color from orange-pink to yellow-green on the sixth day of chicken spoilage [37]. Films made using starch, gelatin, and red radish anthocyanins underwent a visible color transition from orange to grey-purple, perceptible to the naked eye [38]. Considering the prior research findings, it can be deduced that the indicators produced in this study exhibited a highly pronounced color transition, spanning a range of hues from pinkish red to blue to green, depending on the extent of spoilage and the specific day of observation. Consequently, these pH-responsive indicators hold promise for practical utilization in food packaging due to their ease of fabrication, and consistent performance, with the color alterations being easily observable by the naked eye.

### 3.5. Optical Properties

The color changes of the indicator were photographed and are presented in Figure 8. This alteration in color is associated with the deprotonation of anthocyanins caused by TVB-N compounds produced during the degradation process [39]. The color values as determined by the colorimeter are listed in Table 2. The alterations in color were quantified using the (ΔE) value as defined in Equation (ii). The (ΔE) steadily rose in all the examined samples and under various storage conditions, signifying the release of TVB-N as a result of the degradation process. The colorimetric values are mentioned in Table 2. At the outset, the L-value (lightness) exhibited a lower value on day 0, subsequently rising with extended exposure on both day 1 and day 2. As for a-value (redness), it initially displayed prominence but gradually decreased in response to increasing pH levels, attributed to chicken spoilage. However, a-value exhibited a slight increase with further spoilage. In the case of the b-value (blue to yellow), it began with a lower value but increased on day 1 in conjunction with rising pH levels, although experiencing a slight decrease on day 2. The colorimetric measurements taken from the designed indicators consistently matched the visible color alterations observed during the packaging tests, which were also documented through their photograph. These findings exhibited similarities to results observed in other studies as well. For instance, the color changes in these indicators were analogous to those seen in research involving the integration of *Echium amoenum* anthocyanins into bacterial cellulose film [40]. Additionally, studies involving cassava starch film with anthocyanins from *Lycium ruthenicum* Murr [41], the incorporation of aqueous hibiscus extract in starch film [42], and the immobilization of *Bauhinia blakeana* Dunn anthocyanins within chitosan [43], demonstrated similar results.

## 4. Conclusion

This study aimed to develop cost-effective intelligent freshness indicators utilizing agricultural waste (specifically, peels of common plum, *Prunus domestica*) for monitoring the freshness of packaged meat products. Spoilage validation and correlation with indicator color changes were established through the determination of TVB-N, pH, and microbial load in chicken samples.

The sensitivity and reversibility of the indicators were tested by exposure to ammonia and acetic acid vapor. The outcome of the findings indicated that these indicators can efficiently monitor the freshness of packaged chicken meat products from a consumer's standpoint at various stages of spoilage. The indicators performed well with samples ranging from 50 g to 250 g; however, larger samples (500 g and more) exhibited altered color changes due to moisture accumulation and elevated TVB-N. To address this, increasing anthocyanin content in the indicator for larger samples and enhancing moisture resistance can be suggested. Although the indicators stored in the dark remained shelf stable for 8 months, the primary drawback can be the deterioration of the dye. Over time, exposure to strong light, heat, and oxygen may lead to anthocyanin degradation, diminishing its color-changing ability, lifespan, and reliability. To counteract this, methods like acylation or encapsulation could be employed to enhance the stability of the anthocyanin. The smart food spoilage indicators based on anthocyanins hold promise in meeting the rising demand for improved food safety, quality, and sustainability. However, their success is contingent on ongoing research, technological advancements, and evolving consumer preferences favoring sustainable, safe, and high-quality food products.

## Figures and Tables

**Table 1:** TVB-N, APC, and pH values of chicken samples at different days/stages of spoilage.

Day	Total volatile basic nitrogen (TVB-N) (mg/100 g)	Microbial load (aerobic plate count) (log_10_ cfu/g)	pH value
0	3.64 ± 0.39	4.64 ± 0.27	7.03 ± 0.16
1	13.57 ± 0.56	7.72 ± 0.48	7.72 ± 0.08
2	28.17 ± 1.46	9.93 ± 0.32	8.12 ± 0.39

**Table 2:** Colorimetric values of freshness indicators on day 0, day 1, and day 3 of chicken spoilage with a sample size of 50 g.

Indicator no	Red	Blue	Yellow	White
Day 0	4.66 ± 0.47	0.3 ± 0.08	0.06 ± 0.04	0.03 ± 0.04
Day 1	0.36 ± 0.09	0.73 ± 0.16	0.36 ± 0.12	0.16 ± 0.09
Day 2	0.66 ± 0.16	0.56 ± 0.26	0.2 ± 0.08	0.23 ± 0.04

## Data Availability

The data supporting the current study are available from the corresponding author upon request.
